# Application of CD38 monoclonal antibody in kidney disease

**DOI:** 10.3389/fimmu.2024.1382977

**Published:** 2024-05-10

**Authors:** Zhiyi Chen, Qianchun Xu, Zhangfei Shou

**Affiliations:** ^1^ College of Medicine, Zhejiang University, Hangzhou, Zhejiang, China; ^2^ Department of Nephrology, Shulan (Hangzhou) Hospital, Affiliated to Zhejiang Shuren University Shulan International Medical College, Hangzhou, Zhejiang, China; ^3^ Zhejiang University of Traditional Chinese Medicine, Hangzhou, Zhejiang, China

**Keywords:** CD38, daratumumab, lupus nephritis, membranous nephropathy, kidney transplant

## Abstract

CD38 antigen is a glycoprotein that found on the surface of several immune cells, and this property makes its monoclonal antibodies have the effect of targeted elimination of immune cells. Therefore, the CD38 monoclonal antibody (such as daratumumab, Isatuximab) becomes a new treatment option for membranous nephropathy, lupus nephritis, renal transplantation, and other refractory kidney diseases. This review summarizes the application of CD38 monoclonal antibodies in different kidney diseases and highlights future prospects.

## Introduction

The CD38 antigen, a type II transmembrane glycoprotein with ectoenzymatic activity and receptor function, was initially identified in 1980 by S. Schlossman and E.L. Reinherz (1, 2).The CD38 molecule present on the surface of several immune cells because it follows the action of both T-cells and B-cells to trigger its activity as an indicator of cellular activation and differentiation (3, 4). Moreover, It is widely distributed in various tissues of mammals as a multifunctional enzyme for using NAD+ as a substrate to synthetise ADPR and cADPR, mainly expressed in hematopoietic cells, and also has a high level of expression in kidney cells (5).

**Figure 1:**
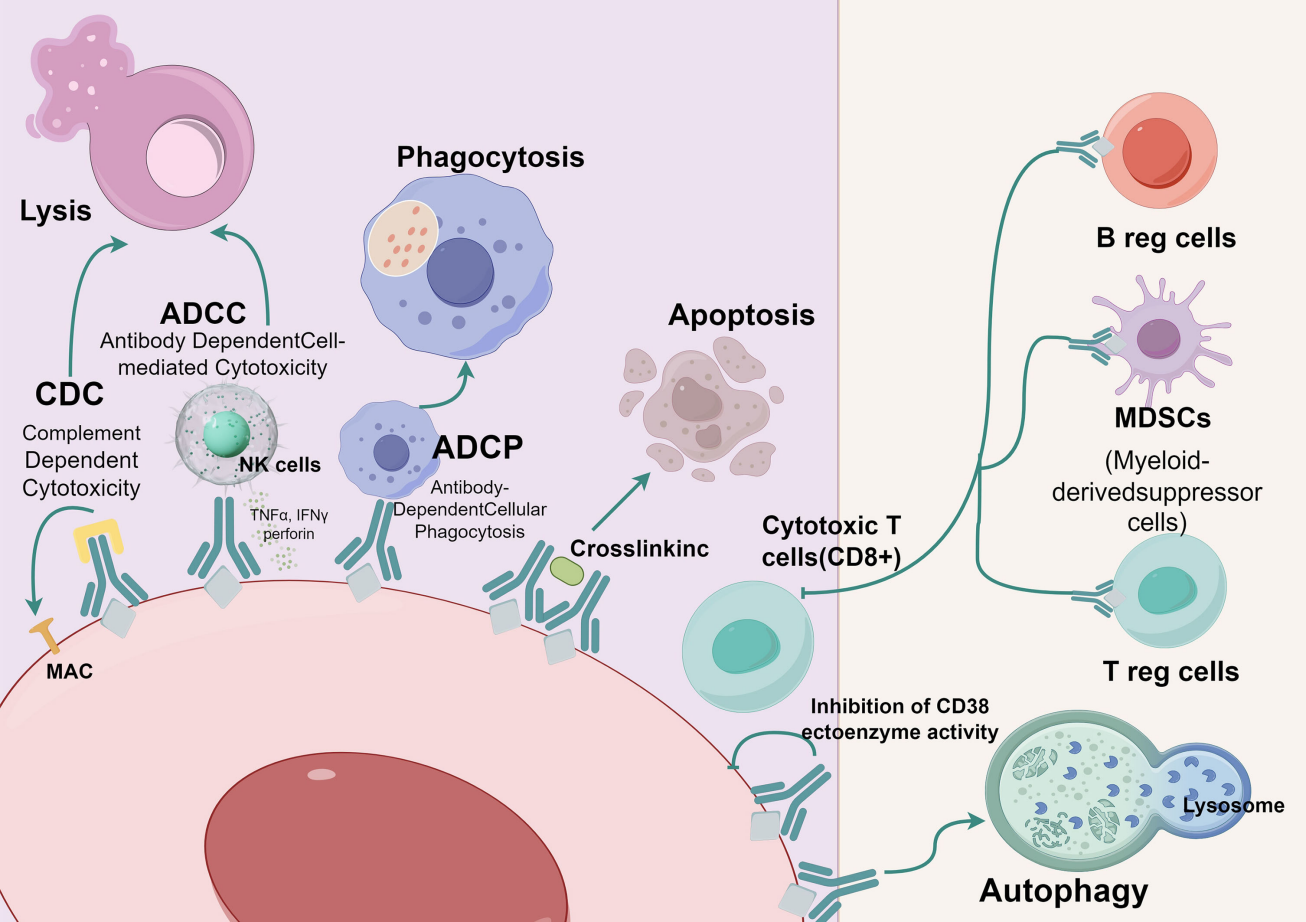
Mechanism of action of anti-CD38. 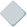 CD38;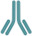 Anti-CD38 (Daratumumab or lsatuximab); CDC, Complement Dependent Cytotoxicity; ADCC, Antibody Dependent Cell-mediated Cytotoxicity; ADCP, Antibody-Dependent Cellular Phagocytosis. (By Figdraw).

CD38 monoclonal antibodies possess a variety of mechanisms for their action. One such antibody, daratumumab, achieves its impacts via Fc-dependent processes, specifically complement-dependent cytotoxicity (CDC), antibody-dependent cellular cytotoxicity (ADCC) (6), antibody-dependent cellular phagocytosis (ADCP) (7), and apoptosis induced by FcγR-mediated crosslinking (8). Another antibody, isatuximab, also induces ADCC, ADCP, and CDC (9). Additionally, isatuximab directly triggers the death of abnormal cells through Fc-dependent mechanisms that involve the apoptotic pathway regulated by caspases and the pathways associated with lysosomal-mediated cell killing (10).. Furthermore, daratumumab exhibits immunomodulatory effects that can eliminate CD38+ immunosuppressive Tregs (11) (**Figure 1**). Preliminary preclinical experiments suggest that isatuximab may also possess these effects (12), but further confirmation is required.

CD38 monoclonal antibodies have traditionally been used to treat hematologic malignancies, especially Multiple Myeloma. However, recent studies propose that these antibodies might also contribute to easing light-chain (AL) amyloidosis (13). Furthermore, they have shown potential as immunologic modulators, offering benefits to patients with autoimmune diseases or those who have undergone organ or stem cell transplantation (14). The research on CD38 has significantly advanced the progress in developing CD38 antibodies. Currently, there are multiple types of CD38 monoclonal antibodies available in the market. Daratumumab, an IgG1-kappa monoclonal antibody, was the pioneering and extensively utilized antibody for treating Multiple Myeloma. Numerous studies have confirmed its effectiveness. In 2020, another IgG1 monoclonal antibody called Isatuximab received approval in the United States (15). Furthermore, there are promising novel antibodies like MOR202 and SAR442085 that hold great potential for future applications (16, 17). Recent research indicates that CD38 inhibitors can treat various refractory kidney conditions (**Table 1**).

**Table 1:** Summary of potential future therapeutic application of CD38 antibody.

Reference	Patient Characteristics	Medication Dosage	Results	Level of evidence
(18)	16-year-old female with MN after GVHD	Daratumumab, 16 mg/kg, once a week for a total of 3 doses	Improvement within 10 weeks	IV
(19)	38-year-old female with refractory aPLA2R-resistant MN	Daratumumab, 16 mg/kg, once a week, later extended to every 2 and 4 weeks	Rapid reduction in aPLA2R level, significant clinical improvement	IV
(20)	38-year-old patient with refractory aPLA2R-resistant MN	Daratumumab, 16 mg/kg, with dexamethasone 40 mg, 8 injections over 2 months	Rapid reduction in aPLA2R levels, significant clinical improvement	IV
(21)	Six patients with multiple drug-resistant refractory LN	Daratumumab,16 mg/kg, once a week for 8 weeks, then every 2 weeks for 8 times, followed by monthly doses	Significant improvement in symptoms and SLE activity in five patients	IV
(22)	50-year-old female with multiple drug-resistant LN	Daratumumab,16 mg/kg, once a week for 4 weeks, then adding Belimumab	Significant symptom improvement, no adverse effects	IV
(23)	49-year-old male post-kidney transplant, CAAR, DSA (+)	Daratumumab, 16 mg/kg, first 2 cycles once a week (8 weeks), then cycles 3-6 every two weeks, followed by monthly doses	Remission of CAAR with only minor infusion reactions	IV
(24)	Two post-kidney transplant patients with high anti-DQ7 dnDSA levels	Daratumumab, 400 mg, once a week	Improved kidney function and a significant reduction in anti-DQ7 DSA levels	IV
(25)	59-year-old male post-kidney transplant, acute AMR	Daratumumab, 16 mg/kg, once a week	Gradual improvement in kidney transplant function, no severe adverse events	IV
(26)	32-year-old male, pre-third kidney transplant, high anti-HLA-I and HLA-II site antibody levels	Daratumumab,400 mg, intravenously 1 day after PP/IVIG, given weekly for 19 weeks	Significant reduction in antibody levels post-transplant, with stable kidney function	IV
(27)	One patient with severe mixed acute kidney and heart rejection due to AMR	Daratumumab,16 mg/kg, combined with eculizumab	Significant improvement in kidney function, substantial decrease in anti-HLA DSAs	IV
(27)	Eight sensitized macaques	Daratumumab (16 mg/kg) and plerixafor (0.24 mg/kg) once a week	Significant reduction in DSA levels and extended initial graft survival time	IV
(28)	54-year-old male with MM and kidney injury post-ABMT	Daratumumab 16mg/kg, once a week	Severe ACR occurred within 48 hours post-surgery	IV
(29)	38-year-old male with C3GN	Daratumumab (16 mg/kg weekly), in combination with dexamethasone and lenalidomide	Stable hematological response, improvement in creatinine and proteinuria	IV
(30)	Ten PGNMID patients and one C3G patient	Daratumumab, 16 mg/kg, once a week for 8 weeks, then once every two weeks for a total of 8 doses	Symptomatic relief in PGNMID patients, no response in C3G patient. Five adverse events.	IV
(31)	Two critically ill patients with ANCA-associated nephritis	Daratumumab, 1800 mg, subcutaneous injection, once a week	Significant clinical improvement in both patients,minor adverse reactions	IV

## Membranous nephropathy

Membranous nephropathy (MN) is a distinct autoimmune disorder that impacts the kidneys. It is primarily caused by antibodies targeting the antiphospholipase A2 receptor (PLA2R), resulting in the accumulation of immune complexes in the glomerular basement membrane (32). Recent research has shown that patients who test positive for PLA2R antibodies experiencing a reduced rate of natural remission compared to individuals who test negative for antibodies (33).Standard therapies, including corticosteroids and cyclophosphamide, may not achieve immunologic remission in approximately 10% of patients with membranous nephropathy associated with anti-phospholipase A2 receptor antibody (aPLA2R). Research has shown that the blend of rituximab, cyclophosphamide, and corticosteroids in a cycle therapy approach has proven to be highly effective. However, it is important to note that there is still a considerable risk of resistance and relapse in about 20% to 30% of cases (34). To address this challenge, there is a need for fresh therapeutic alternatives for patients with membranous nephropathy who do not respond to rituximab. Long-lived plasma cells (CD19^-^CD20^-^CD38CD138) in inflamed tissues and bone marrow are crucial for sustained antibody production in MN (35). Current treatments may only reduce their numbers in secondary lymphoid organs. CD38, highly expressed in spleen and bone marrow plasma cells, is targeted by potential therapies (36). This approach offers hope for improving MN prognosis.

Benoit and colleagues documented a case involving a 16-year-old white woman who developed nephrotic syndrome after having graft-versus-host disease (GVHD). The patient was later diagnosed with membranous nephropathy (MN) and tested negative for anti-pla2R antibodies in both serum and kidney tissue. Despite receiving treatment with ibrutinib and a combination of rituximab, tacrolimus, and steroids for 8 months, her kidney condition did not improve. Because other treatments are not suitable for her condition, contemplated plasma cell depletion therapy with Daratumumab. The patient was given three doses of Daratumumab (16 mg/kg) at weeks 1, 4, and 17. By week 10, the patient’s nephrotic syndrome had resolved, leading to the discontinuation of tacrolimus and steroids at week 16. At this point, her kidney disease was almost completely in remission. IgG levels remained within normal range during the 6-month follow-up. It is unclear whether the rapid response can be attributed to the previous 8 months of treatment. The successful removal of CD38 suggests that resident plasma cells producing allogeneic antibodies may significantly contribute to the complexity of treating this specific ailment. In conclusion, Daratumumab may offer a promising new treatment option for individuals unresponsive to standard treatments (18).

A case study conducted by Didier Ducloux et al. showcased a patient who showed no response to mycophenolate mofetil/prednisolone and rituximab treatment, and had adverse reactions to cyclophosphamide and bortezomib/dexamethasone. However, when the patient was switched to daratumumab (administered intravenously at a dose of 16 mg/kg per week), there was a swift decline in aPLA2R levels, accompanied by significant clinical improvement. The break between daratumumab doses was gradually extended to 2 and then 4 weeks, Leading to a gradual rise in aPLA2R levels. Additionally, the patient’s naive B cells increased significantly and plasma cells became undetectable. Subsequently, daratumumab was discontinued and rituximab (administered at a dose of 2 g) was reintroduced. This led to another rapid and persistent decrease in aPLA2R levels. After receiving rituximab treatment for 7 months, the patient partially achieved clinical remission and maintained stable kidney function (19).In another study conducted by Andreas Kronbichler et al., they also documented the utilization of daratumumab therapy in a 38-year-old patient with multidrug-resistant aPLA2R refractory MN. The short-term results are consistent with the above experiments, further supporting the potential efficacy of daratumumab in such cases (20).

FelzarTamab (CD38 antibody) is currently being tested in Minnesota through several open-label multicenter trials. These trials are specifically focused on adult patients with membranous nephropathy who have anti-PLA2R antibodies (NCT04145440, NCT04733040) and patients with membranous nephropathy who have not responded to anti-CD20 targeted therapy (NCT04893096).

In summary, numerous studies have indicated that the administration of a standard dosage of anti-CD38 could potentially contribute to the treatment of PLA2R-positive MN, particularly for patients who have not responded well to conventional MN therapies. This treatment has demonstrated the ability to induce rapid clinical and immunologic remission, although its long-term effectiveness is not so ideal. However, as an anti-plasma therapy, anti-CD38 may exhibit even greater efficacy when combine with anti-B cell therapy. For instance, researchers have explored the combination of daratumumab and obinutuzumab as a strategy for achieving prolonged alleviation in specific types of nephrotic syndrome (37). It is important to conduct further investigations in order to establish the optimal treatment approach.

## Lupus nephritis

Systemic lupus erythematosus (SLE) is a persistent autoimmune condition predominantly affecting women during their reproductive years. It can lead to harm in multiple organs, including the kidneys, blood, and nervous system (38). In individuals with SLE, lupus nephritis (LN) stands out as the most prevalent and severe renal manifestation, impacting more than half of the patients (39). While traditional immunosuppressive treatments have shown effectiveness in a significant portion of patients, there remains a subset of individuals who do not respond well to these therapies. Consequently, there is a continued need for the development of new treatment options.

SLE is characterized by an overactive immune response, involving B cells and plasma cells that produce excessive amounts of autoantibodies. This immune dysregulation can result in damage to cells and tissues (35, 40, 41). In contrast to traditional immunosuppressive therapies aimed at depleting B cells, using CD38-targeted antibodies to selectively eliminate plasma cells secreting pathogenic antibodies has emerged as a novel option.

Recent studies reveal an increased count of transitional B cells (CD38+IgM+) in individuals with SLE, playing a pivotal role in the maturation of B cells (42).. Furthermore, preclinical models have confirmed the presence of Prolonged plasma cells in lupus patients (43). These plasma cells are believed to contribute significantly to the progression of autoimmunity. Additionally, it has been observed that lupus nephritis (LN) patients have an increased number of macrophages, whose CD38 expression is specifically activated and up-regulated, suggesting that CD38 might contribute to the regulation of lupus nephritis (LN) development and progression by influencing immune responses (44). CD38 monoclonal antibodies can effectively eliminate plasma cells and modulate immune responses, therefore holds potential benefits for LN patients.

In a recent investigation conducted by Dario Roccatello et al., six cases of refractory lupus nephritis (LN) were examined. The six patients are not responsive to standard treatments containing mycophenolate mofetil (MMF) and cyclophosphamide/azathioprine. Subsequently they are treated with intravenous daratumumab, (16 mg/kg weekly infusions for 8 weeks, followed by 8 biweekly infusions and then 8 monthly infusions).Out of the six patients, five exhibited substantial amelioration in both symptoms and disease activity. The mean disease activity, measured by the SLE Disease Activity 2000 index, the score reduced from 10.8 prior to treatment to 3.6 after 12 months of treatment (P = 0.03). Additionally, there was a significant decrease in proteinuria levels, dropping from 5.6 g per 24 hours to 0.8 g per 24 hours (P = 0.0010), and serum creatinine levels decreased from 2.3 mg/dl to 1.5 mg/dl (P = 0.98) following one year of treatment. The study also found changes in various biomarkers associated with LN. B cell maturation antigen, soluble CD163 levels, and interferon-gamma levels, decreased, while C4 and interleukin-10 levels increased in response to daratumumab treatment. Meanwhile, seroconversion of anti-double-stranded DNA antibodies appear in these patients (anti-dsDNA decreased from 157 IU/L to 12 IU/L (P = 0.03)).These findings suggest that daratumumab could offer new possibilities for managing resistant lupus nephritis LN (21).

Lennard Ostendorf and colleagues administered daratumumab to address severe and life-threatening cases of systemic lupus erythematosus (SLE) in two patients. One of the patients was a LN patient with several complications and previous therapy containing cyclophosphamide and bortezomib failed. After 4 months of treatment with daratumumab (16 mg/kg, once a week for 4 weeks), an anti-B cell antibody belimumab was added to maintain the efficacy. Over the 12-month follow-up duration, significant improvements were observed in the patient. Urine protein decreased from more than 6 g/day to about 1 g/day, Serum creatinine levels returned to normal as well as serological analysis showed a marked reduction in anti-double-stranded DNA antibodies. Although both patients experienced a marked decline in IgG levels, one’s remained normal and the other’s stabilized after two doses of intravenous immune globulin, with no infections observed. Importantly, no adverse effects were reported. Researchers also discovered that in individuals with lupus, CD38 expression extends beyond plasma cells to include plasma blasts, mature B cells, and dendritic cells resembling plasma cells. Additionally, they observed an expansion of CD38-expressing T cells. As a result, daratumumab has shown a remarkable impact in treating lupus patients (22).

Mayo Clinic is currently conducting a Phase 2 clinical trial (NCT04868838) to assess the effectiveness and safety of Daratumumab in patients diagnosed with active LN. The trial involves administering Daratumumab once weekly for 8 weeks, then transitioning to once every 2 weeks for an additional 8 doses (with a variation of +/- 4 days). Patients will be monitored for a total of 24 month. This study aims to provide comprehensive insights into the potential benefits of daratumumab as a treatment option for lupus nephritis.

The short-term treatment of LN necessitates an adequate duration (6-12 months) to achieve complete remission of kidney function. Prolonged ineffective treatment, can result in chronic kidney damage (45). Striking the right balance between disease activity, organ damage, and immunosuppression is crucial for effective management. The use of daratumumab for LN has shown promising results in these case reports for the patients experienced significant improvement in clinical symptoms without notable adverse reactions. However, further evidence from multicenter open-label trials is necessary to confirm the safety and efficacy of daratumumab in LN patients. Additionally, it is important to acknowledge that daratumumab only provides temporary depletion of plasma cells and should be used in conjunction with other immunosuppressants to inhibit the development of transforming autoreactive B cell precursors into autoreactive plasma cells.

## Kidney transplantation

Kidney transplantation is widely recognized as the treatment with the highest efficacy for individuals with end-stage renal disease (ESRD). To ensure the success of the transplant surgery and minimize the chances of acute rejection, patients typically need induced immunosuppressive therapy. A significant factor contributing to the dysfunction of kidney grafts is antibody-mediated rejection (AMR), mainly induced by donor-specific antibodies (DSA) generated by long-lived plasma cells (46).There is a lack of effective immunosuppressive agents for the treatment of AMR. Previous attempts with the anti-CD20 agent rituximab and the proteasome inhibitor bortezomib frequently fail (47). On the other hand, highly sensitized patients who require pre-transplant desensitization face a challenging situation as current treatments such as high-dose Intravenous Immunoglobulin(IVIG) and plasmapheresis have limited efficacy (48). However, there is potential in exploring CD38 targeting antibodies as a new approach. These antibodies not only have the ability to eliminate malignant plasma cells (PC), but may also lead to a decrease in the generation of autoantibodies or anti-HLA antibodies in autoimmune diseases, thus reducing the antibody-dependent effector mechanism (27). This suggests that CD38 targeting antibodies may present a hopeful choice for addressing AMR and pre-transplant desensitization.

Konstantin and his colleagues highlighted a remarkable case of a 49-year-old male who had received a kidney transplantation and was later diagnosed with smoldering myeloma. The patient presented with chronic active AMR and tested DSA-positive. To address this complex situation, the patient was treated with intravenous daratumumab. The treatment regimen consisted of a weekly dose of 16mg/kg for the first two cycles (8 weeks), followed by biweekly doses for the next 16 weeks (cycles 3-6), and then monthly doses thereafter. Consequently, there was a notable reduction in the patient’s natural killer (NK) cells in both blood and the transplanted kidney. Additionally, the serum levels of donor-specific antibodies disappeared. Follow-up biopsies of the transplanted kidney revealed a remarkable improvement in microvascular inflammation and rejection-associated molecular classifiers. Furthermore, the patient’s kidney function remained stable throughout this period. It is worth noting that apart from a mild infusion reaction characterized by allergic rhinitis during the initial administration of daratumumab, no other adverse effects were detected (23). Lan Zhu et al. have developed an innovative treatment approach for AMR in kidney transplant recipients. Their regimen involves early intensive treatment with daratumumab, along with plasmapheresis (PP) or IVIG, followed by maintenance therapy with solely daratumumab. Two patients with confirmed AMR and high levels of anti-DQ7 dnDSA were treated using this regimen. The results were promising, as both patients experienced improvements in renal function and a notably decrease in anti-DQ7 DSA levels (from approximately 20,000 to 5,000 MFI value) shortly after receiving the treatment. One patient’s condition remained stable for 20 months, while the other patient experienced a decline after one year due to acute T cell-mediated rejection. The treatment was well-tolerated, only mild symptoms resembling the flu were reported after the first dose of daratumumab. No other adverse effects were observed. These findings verified the short-term effectiveness of daratumumab in interfering with AMR (24).

In a case study, Spica and his teammates reported a 59-year-old man who underwent a kidney transplant from a living donor with an ABO-incompatible blood type. The patient experienced acute AMR after the transplant. The standard combined therapy of anti-human t-lymphocyte globulin, methylprednisolone, and Eculizumab was not effective. To mitigate the risk of prolonged reliance on immunoadsorption and the potential for graft failure, medical team decided to administer Daratumumab as a rescue treatment on postoperative day 30. The patient received infusions of Daratumumab at a dosage of 16mg/kg once weekly. Remarkably, the levels of blood group antibodies (anti-A IgM and IgG) quickly decreased and remained low without the need for further immunoadsorption. Additionally, the patient’s kidney graft function gradually improved without any severe adverse events. The case suggest that Daratumumab may be a valuable replacement therapy for managing acute AMR in similar cases (25).

Zhao et al. recently published a case study involving a 32-year-old man who underwent his third kidney transplant. This patient had a significant amount of antibodies targeting the HLA-I and HLA-II loci and his cPRA score exceeding 99%. To address this challenge, a two-phase desensitization treatment plan was implemented. In the first phase, the patient received rituximab as an initial treatment, followed by PP/IVIG plus daratumumab (400 mg, intravenously) one day after PP/IVIG. This combination therapy was administered weekly for a total of 19 weeks. This approach successfully reduced the levels of antibodies and significantly lowered the cPRA score. The second phase of treatment aimed to sustain the decline in antibodies while awaiting a compatible donor. Eventually, the patient received a kidney transplantation with an HLA mismatch grade of 4, and the concentrations of donor-specific antibodies were greatly reduced following the transplantation. The patient experienced a smooth recovery after the transplantation, and the graft function remained stable throughout the one-year follow-up period. Importantly, minimal adverse effects were observed from the desensitization treatment. These promising results suggest that this approach holds great potential for highly presensitized patients in need of kidney transplantation though further clinical studies are necessary to validate this desensitization strategy in similar cases (26).

Kwu et al. conducting a clinical case involving a patient with AMR who was treated with a combinnation of eculizumab and Daratumumab. The patient had a severe mixed acute renal and cardiac rejection. After the treatment, there was a significant improvement in kidney function, with serum creatinine levels decreasing to 350 mmol/L. Furthermore, two class 2 anti-HLA DSAs decreased significantly and became undetectable through circulating PC analysis (27). Meanwhile, to investigate the impact of desensitization treatment and AMR before kidney transplantation, they performed experiments on animals that had been sensitized prior to the transplant using Daratumumab and pleriafor (anti-cxcr 4). The results showed that the animals receiving daratumumab experienced a significant decrease in DSA levels compared to the control group (57.9% vs 13%; P <0.05). Additionally, these animals had extended initial graft survival (28 vs 5.2 days, P<0.01).However, it was observed that all recipients exhibited a swift resurgence of antibodies and T cell-mediated rejection after some time. This could be attributed to the decrease in regulatory B and T cells and the faster appearance of activated T cells in the animals treated with Daratumumab (27).

The same condition occurred in another case. Scalzo et al. described a 54 years old patient who had multiple myeloma. The patient experienced kidney injury and underwent autologous bone marrow transplantation (ABMT). After 3 months, the patient received maintenance therapy with Daratumumab administered at a dosage of 16 mg/kg per week. Despite having a low immune risk of matching, including being a white individual with a living donor renal receptor, brief cold ischemia duration, and absence anti-HLA antibodies (DSAs or other), the patient developed intense acute T cell-mediated rejection occurred within a 48-hour timeframe after the surgery. The duration of Daratumumab’s effectiveness varied between 9 and 27 days, and in this particular case, the patient was administered a dose of Daratumumab two weeks prior to the transplant but did not receive any further doses afterwards. While it is possible that the patient’s immune response, specifically the b-cell and memory T cell responses, could have contributed to the negative outcome, researchers suggest that administering the drug within 27 days or less before transplantation may have adverse effects on the transplanted organ. Additionally, it is worth mentioning that the patient also developed hypogammaglobulinemia during treatment, although it is uncertain whether this phenomenon was induced by daratumumab (28).

Currently, there are multiple ongoing clinical trials investigating the efficacy of CD38 monoclonal antibodiessuch as daratumumab, isatuximab, and felzartamab in the context of kidney transplantation. These trials specifically aim to diminish the incidence of antibody-mediated rejection (NCT05913596 and NCT05021484) or to Investigate the potential advantages for patients who are sensitized and in need of a kidney transplantation (NCT04827979, NCT05145296, NCT04204980, and NCT04294459). Multiple studies are also being conducted to investigate the safety and effectiveness of daratumumab (or chimeric mouse/human isatuxumab).

Daratumumab has shown great potential in addressing AMR. Numerous case studies have highlighted the ability of daratumumab to decrease antibody levels, stabilize kidney function, and enhance overall outcomes with minimal adverse effects. Additionally, animal trials have provided further evidence of its efficacy in reducing DSA levels and prolonging the lifespan of transplanted organs. Moreover, an innovative desensitization approach utilizing daratumumab has demonstrated promising results in highly sensitized patients who are on the kidney transplantation waiting list. Nevertheless, apprehensions arise regarding the safety of conducting solid-organ transplants in patients who are on daratumumab due to the swift resurgence of antibodies and T cell-mediated rejection it can induce. Therefore, further research is necessary to evaluate the advantages and disadvantages of CD38 antibody therapy in this context.

## Other refractory kidney diseases

Due to its ability to increase the number of T cells in the bone marrow and blood, thereby enhancing the immune response against malignant clone cells, anti-CD38 antibodies may also be used for other kidney diseases (49). Esposito et al. reported a case of 38-year-old man who was diagnosed with C3 glomerulonephritis (C3GN) and required Substitution therapy. After the initial treatment proves ineffective, daratumumab was used and lead to the gradual enhancement of the patient’s renal function and renal biopsy, 10  months later even hemodialysis was discontinued (29). Zand et al. conducted a clinical trial of 10 proliferative glomerulonephritis with monoclonal Ig deposits (PGNMID) patients, they were treated with at least one dose of daratumumab. Throughout the 12-month follow-up period, 4 of them had a complete response while the other 6 achieved a partial response (30). The validity was reproduced in Almaani et al.’s study (50). Relevant clinical trials are ongoing(NCT05654506).Lennard Ostendorf et al. used Daratumumab to rescue two patients with anti-neutrophil cytoplasmic antibody (ANCA) -related nephritis (31). Ongoing clinical trials are exploring other applications of CD38 antibody containing IgA nephropathy (NCT05065970) and even metastatic renal cell carcinoma (NCT03473730).The study( NCT05704400 )is researching the effectiveness of the combination therapy of CD20 monoclonal antibody and daratumumab monoclonal antibody in the treatment of pediatric multidrug-resistant nephrotic syndrome (**Table 2**).

**Table 2:** Summary of ongoing trials of CD38 antibodies.

NCT number	Drugs	Disease or Conditions	Phase
NCT04145440	Felzartamab	PLA2R-positive MN	1 and 2
NCT04733040	FelzarTamab	PLA2R-positive MN	2
NCT04893096	FelzarTamab	Refractory MN	2
NCT04868838	Daratumumab	Lupus Nephritis	2
NCT05913596	Daratumumab	Antibody-mediated Rejection of Kidney Tranplantation	–
NCT05021484	Felzartamab	Antibody-mediated Rejection of Kidney Tranplantation	2
NCT04827979	daratumumab and belatacept	Highly Sensitized Prospective Kidney Transplant Recipients	1 and 2
NCT05145296	daratumumab and belatacept	Highly Sensitized Prospective Kidney Transplant Recipients	–
NCT04204980	Daratumumab	Kidney Transplant Rejection	1 and 2
NCT04294459	Isatuximab and others	Kidney Transplant Rejection	1 and 2
NCT05654506	Daratumumab	PGNMID	2
NCT05065970	Felzartamab	IgA Nephropathy	2
NCT03473730	Daratumumab	Clear Cell Renal Cell Carcinoma	1
NCT05704400	Rituximab and Daratumumab	Nephrotic Syndrome	1

PLA2R, phospholipase A2 receptor; MN, membranous nephropathy; PGNMID, proliferative glomerulonephritis with monoclonal immunoglobulin deposits.

Some studies also discusses how blocking CD38 can potentially reduce kidney injury and explores the therapeutic potential of CD38 monoclonal antibodies for kidney diseases like allergic purpura nephritis and granulomatous polyangiitis (51–53). In a word the potential of the CD38 monoclonal antibodies in kidney diseases remains to be exploited.

## Adverse effects

Common adverse reactions associated with the administration of CD38 monoclonal antibodies frequently manifest as infusion-related symptoms, including but not limited to dyspnea, pulmonary edema, myocardial infarction, cough, chills, throat irritation, nausea, and vomiting. Hypogammaglobulinemia may occur initially, but the IgG levels tend to remain stable, and no severe outcomes were observed in the cases above. To mitigate the incidence and severity of these adverse reactions, medical practitioners may consider pre-treatment with intravenous corticosteroids and appropriate fluid supplementation. Rigorous monitoring is imperative throughout the medication’s administration, and, in the presence of adverse reactions, it is prudent to gradually reduce the infusion rate or temporarily suspend it until the untoward effects abate. Resumption of the medication should be executed cautiously upon the stabilization of the patient’s condition.

Furthermore, when addressing infectious complications arising from CD38 monoclonal antibodies therapy, the judicious use of antibiotics may be warranted. It should be emphasized that, in comparison to alternative immunosuppressants, the incidence of short-term adverse reactions with CD38 monoclonal antibodies appears to be consistent. Nevertheless, the need for further randomized controlled trials (RCTs) and comprehensive extended follow-up period studies is imperative to comprehensively elucidate potential long-term complications.

## Discussion

Treating kidney disease necessitates time for full renal function remission, yet extended ineffective therapy can result in chronic kidney damage. Achieving the right equilibrium among disease control, organ preservation, and immunosuppression is paramount (45). CD38 monoclonal antibodies offers advantages such as high efficacy, immunomodulatory effects, applicability in various treatment stages, and generally lower adverse reactions compared to some other therapies. It represents a viable and secure choice for individuals with kidney disease. especially those resistant to other treatments.

But there are some concerns. First, CD38 monoclonal antibodies’s ability to deplete plasma cells is temporary. To effectively inhibit the transformation of auto-reactive B cell precursors into auto-reactive plasma cells, combination with additional immunosuppressants is needed (54). Secondly, Certain CD38 monoclonal antibodies recipients experienced rapid antibody resurgence and T-cell-mediated rejection which may introduce additional interaction mechanisms that could induce cellular rejection responses toward transplanted organs. Further randomized controlled trials (RCTs) and long-term monitoring are required to investigate its safety profile.

Considering the validation of CD38 antibody safety and individual variability in therapeutic efficacy, establishing a rational monitoring strategy is paramount. In clinical practice involving such therapeutics, attention should be directed toward blood drug concentrations, assessments of renal function (regular urinalysis including urinary protein, hematuria, and serum creatinine, as well as measurement of eGFR), cytometry-based immune monitoring including plasma cell counts and variations in NK cell counts within PBMC (Peripheral Blood Mononuclear Cell), serum immunoglobulin titers, adverse reaction events, and infection indicators. Additionally, kidney transplant candidates and recipients should pay particular attention to intra-patient variability of calculated panel-reactive antibody (cPRA), mean fluorescence intensity (MFI) of anti-HLA antibodies, and variations in ABO antibody titers. Monitoring changes in ds-DNA is of utmost importance for LN patients. The monitoring should be sustained for at least 6 months, with 1 to 2 years of follow-up recommended if conditions permit.

Finally, while current research and clinical cases indicate favorable tolerance among the majority of patients receiving CD38 monoclonal antibodies, a small percentage may still encounter severe complications. Therefore, close monitoring of patients’ vital signs and reactions is necessary during infusion. CD38 monoclonal antibodies offer hope for refractory kidney diseases and an alternative for sensitized kidney transplant candidates. Despite their efficacy, the high cost limits accessibility. Further research is needed to explore their effectiveness, optimal use, and potential to replace current treatments. Anticipated multicenter trials will contribute to a solid foundation for their application in kidney disease treatment.

## Author contributions

ZC: Writing – original draft. QX: Writing – original draft. ZS: Writing – review & editing.
